# L-tryptophan and copper interactions linked to reduced colibactin genotoxicity in *pks+ Escherichia coli*

**DOI:** 10.1128/msystems.00992-24

**Published:** 2024-09-12

**Authors:** Charlie Bayne, Magali Boutard, Tom Zaplana, Andrew C. Tolonen

**Affiliations:** 1 Department of Pharmacology, University of California, San Diego, California, USA; 2 Génomique Métabolique, Genoscope, Institut François Jacob, CEA, CNRS, Univ Evry, Université Paris-Saclay, Evry, France; University of Massachusetts Amherst, Amherst, Massachusetts, USA

**Keywords:** colibactin, *Escherichia coli*, metabolomics

## Abstract

Colibactin, a nonribosomal peptide/polyketide produced by *pks+ Enterobacteriaceae*, is a virulence factor and putative carcinogen that damages DNA by interstrand crosslinking (ICL). While the *clb* genes for colibactin biosynthesis have been identified, studies are needed to elucidate the mechanisms regulating colibactin production and activity. Here we perform untargeted metabolomics of *pks+ Escherichia* coli cultures to identify L-tryptophan as a candidate repressor of colibactin activity. When *pks+ E. coli* is grown in a minimal medium supplemented with L-tryptophan *in vitro* ICL of plasmid DNA is reduced by >80%. L-tryptophan does not affect the transcription of *clb* genes but protects from copper toxicity and triggers the expression of genes to export copper to the periplasm where copper can directly inhibit the ClbP peptidase domain. Thus, L-tryptophan and copper interact and repress colibactin activity, potentially reducing its carcinogenic effects in the intestine.

**IMPORTANCE:**

Colibactin is a small molecule produced by *pks*+ *Enterobacteriaceae* that damages DNA, leading to oncogenic mutations in human genomes. Colibactin-producing *Escherichia coli* (*pks*+) cells promote tumorigenesis in mouse models of colorectal cancer (CRC) and are elevated in abundance in CRC patient biopsies, making it important to identify the regulatory systems governing colibactin production. Here, we apply a systems biology approach to explore metabolite repression of colibactin production in *pks*+ *E. coli*. We identify L-tryptophan as a repressor of colibactin genotoxicity that stimulates the expression of genes to export copper to the periplasm where it can inhibit ClbP, the colibactin-activating peptidase. These results work toward an antibiotic-sparing, prophylactic strategy to inhibit colibactin genotoxicity and its tumorigenic effects in the intestine.

## INTRODUCTION

The human gut microbiome produces numerous metabolites that affect the initiation and progression of disease. Among these metabolites, colibactin is a nonribosomal peptide/polyketide hybrid natural product synthesized by various *Enterobacteriaceae,* including a subset of isolates belonging to the genera *Escherichia*, *Klebsiella*, and *Citrobacter* (1). Colibactin is a procarcinogen that alkylates DNA, causing interstrand crosslinks (ICLs), double-strand breaks, and increased levels of gamma-H2aX in eukaryotic cells (2, 3). The genotoxic effects of colibactin result in host cell blockage at the G2/M transition (2), leading to megalocytosis and impeding regeneration of the intestinal epithelium, which could increase bacterial fitness by prolonging intestinal colonization (4). Mouse models of sepsis (5) and a rat model of meningitis (6) established colibactin as a virulence factor of extraintestinal pathogenic *Escherichia coli* that are associated with urinary tract infections, neonatal meningitis, sepsis, and pneumonia (7). Evidence increasingly supports that colibactin has a role in the etiology of colorectal cancer (CRC): colibactin-producing *E. coli* cells promote tumorigenesis in mouse models of CRC and are elevated in abundance in CRC patient biopsies (60%) relative to non-CRC controls (20%) (8, 9), and the colibactin mutational signature is present in human cancer genomes (10, 11).

Biosynthesis of colibactin requires a 54 kb *pks* island containing 19 *clb* genes (*clbA* to *clbS*), encoding nonribosomal peptide synthases (NRPS), polyketide synthases (PKS), NRPS/PKS megasynthases, an efflux pump, and accessory and processing enzymes (12). Synthesis of colibactin involves multimodular assembly of precolibactin by NRPS/PKS. Ultimately, active colibactin is produced using a prodrug resistance mechanism based on the cleavage of the N-terminal myristoyl-asparagine from precolibactin by ClbP, a periplasmic peptidase anchored in the inner membrane (13, 14). While colibactin abundance has eluded direct measurement due to its instability, studies have made advances to elucidate the structural basis of colibactin and its DNA cross-linking activity (15, 16). In addition, expression of the *clb* genes has been shown to be regulated by environmental factors including iron (17), polyamines (18), and carbon sources (19).

The goal of this study is to identify metabolites that repress or inhibit colibactin activity using the model *pks+ E. coli* ATCC 25922, a strain encoding a *clb* cluster with 99%–100% identity to those of uropathogenic strain CFT073, probiotic Nissle1917, and newborn meningitis strain IHE3034 (20). The cellular potential to produce mature colibactin from precolibactin can be quantified using a fluorogenic probe of ClbP activity (21, 22). Here, we used an activity-based probe that releases fluorescent 7-hydroxy-4-methylcoumarin (7H4M) upon cleavage by ClbP (21) to identify differences in ClbP activity in *E. coli* ATCC 25922 growing in different conditions. We perform a time series of untargeted metabolomics in growth conditions with different ClbP activities, revealing tryptophan as a potential repressor. We examine how L-tryptophan supplementation in *E. coli* ATCC 25922 cultures affects colibactin genotoxicity and genome-wide transcription, identifying a link between L-tryptophan and copper export. We therefore investigate how copper affects ClbP activity *in vivo* and the activity of the purified ClbP peptidase domain. Together, our experiments support that L-tryptophan is linked to copper export and reduced ClbP activity, representing a potential strategy to reduce colibactin genotoxicity in the intestine.

## RESULTS

### ClbP activity varies across growth conditions

**Fig 1:**
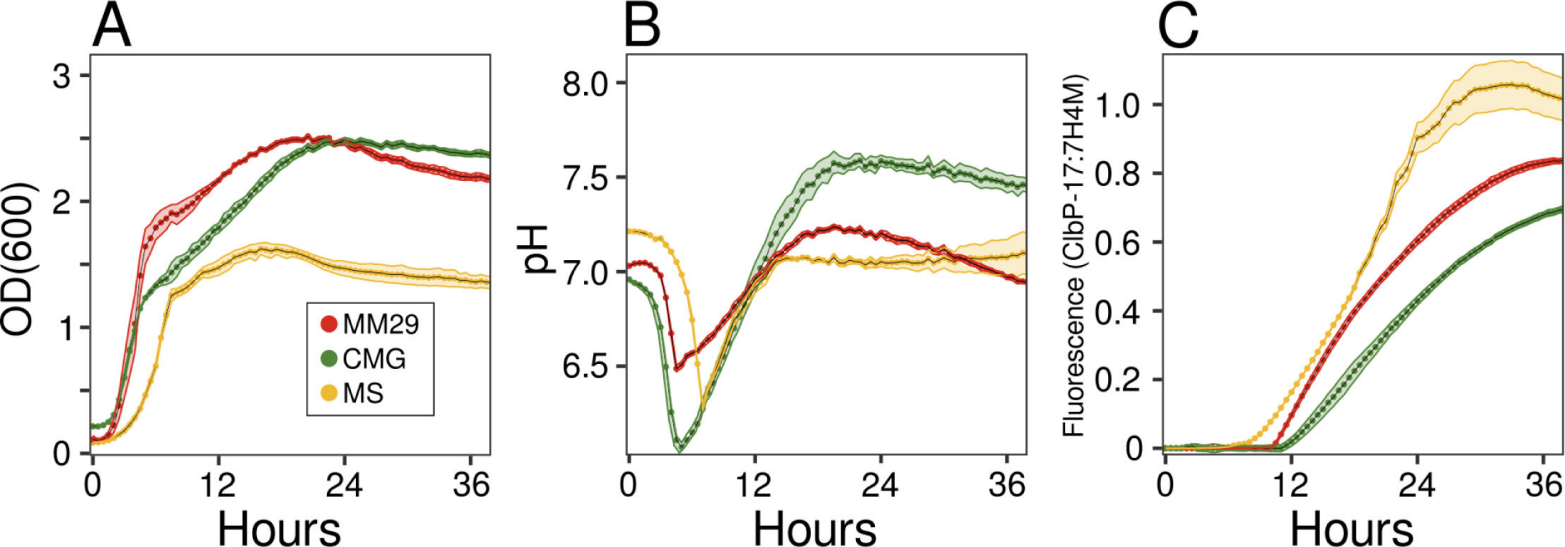
Cultivation of *E. coli* ATCC 25922 in different media. The (**A**) growth, (**B**) pH, and (**C**) ClbP-17:7H4M fluorescence of cultures growing in MS (yellow), MM29 (red), or CMG (green) media. Cultures contained 2 µM BCECF and 100 µM of either ClbP-17 or 7H4M. Growth was measured as optical density at 600 nm (OD_600_). Culture pH was measured as the ratio of the fluorescence of BCECF at the pH-sensitive point (485 ± 15 nm excitation; 540 ± 20 nm emission) relative to the pH-insensitive isosbestic point (450 ± 15 nm excitation; 540 ± 20 nm emission). Fluorescence of ClbP-17 and 7H4M were measured as 360 ± 40 nm excitation, and 440 ± 20 nm emission. ClbP-17:7H4M is the fluorescence of ClbP-17 divided by the median 7H4M fluorescence of replicate cultures. Data points are means of triplicate cultures with shaded areas showing ±SD. BCECF, [2,7-bis-(2-carboxyethyl)5-(and-6)-carboxyfluorescein]; SD, standard deviation.

Expression of colibactin genes (*clb* genes) in *pks+ E. coli* is affected by growth state and medium composition (23). For example, transcription of *clbR,* encoding a transcriptional activator of colibactin genes, is elevated in minimal (M9) medium relative to rich media (24). We examined the activity of ClbP, the periplasmic colibactin-activating peptidase, in *pks+ E. coli* ATCC 25922 growing in different media: chopped meat glucose (CMG) and mega medium (MM29) are rich media and MS is a defined, minimal medium (Fig. 1A). To quantify ClbP activity, we synthesized a ClbP activity-based probe (21) (Fig. S1), hereafter ClbP-17. Upon cleavage by ClbP, ClbP-17 releases 7H4M, which can be monitored by fluorescence (Fig. S2A). We first established that ClbP-17 fluorescence is specific to *pks+ E. coli* (Fig. S2B through E) and that the addition of ClbP-17 or 7H4M did not affect *E. coli* growth in CMG, MM29, or MS media (Fig. S3).

During our tests of ClbP-17, we observed that the fluorescence of 7H4M is sensitive to pH in the physiological range with a 15-fold variation in fluorescence between pH 6 and 8 (Fig. S4). Differences in pH during *E. coli* growth would thus influence fluorescence measurements using ClbP-17. The pH in cultures can be measured in real-time based on the fluorescence of BCECF [2,7-bis-(2-carboxyethyl)5-(and 6)-carboxyfluorescein] (25, 26). We confirmed that BCECF does not affect the growth of *E. coli* ATCC 25922 (Fig. S3) and developed this method to quantify the pH dynamics of ATCC 25922 growing in MS, MM29, and CMG media (Fig. S5). The pH of cultures in all three media initially decreased as the cells produced acids during log-phase growth, then increased as cells entered the stationary phase (Fig. 1B). The greatest pH increase occurred in the CMG medium, likely due to enhanced bacterial liberation of ammonia associated with increased amino acid metabolism. Changes in pH during growth of *E. coli* cultures mirrored changes in 7H4M fluorescence (Fig. S6), confirming that growth-associated changes in pH drive variation in 7H4M fluorescence. To control for variation in 7H4M fluorescence, we thus present ClbP-17 measurements as the ClbP-17:7H4M ratio by dividing ClbP-17 fluorescence by the median 7H4M fluorescence of triplicate samples incubated under the same conditions in adjacent wells.

Growth rate and cell densities in *E. coli* ATCC 25922 cultures were lower in MS than in MM29 or CMG media (Fig. 1A). In contrast, ClbP-17:7H4M fluorescence was highest in MS medium, intermediate in MM29, and lowest in CMG medium (Fig. 1C). This inverse relationship between growth and ClbP-17:7H4M fluorescence in ATCC 25922 reflects prior results that *clbR* transcription is higher in minimal medium (24). As an expression of *clb* genes is repressed by the carbon storage regulatory protein CsrA (27, 28), we hypothesized that a metabolite that represses ClbP in the CMG medium is absent in the defined medium and present in lower quantities in the MM29 medium.

### Metabolomics identifies tryptophan as a potential ClbP repressor

**Fig 2:**
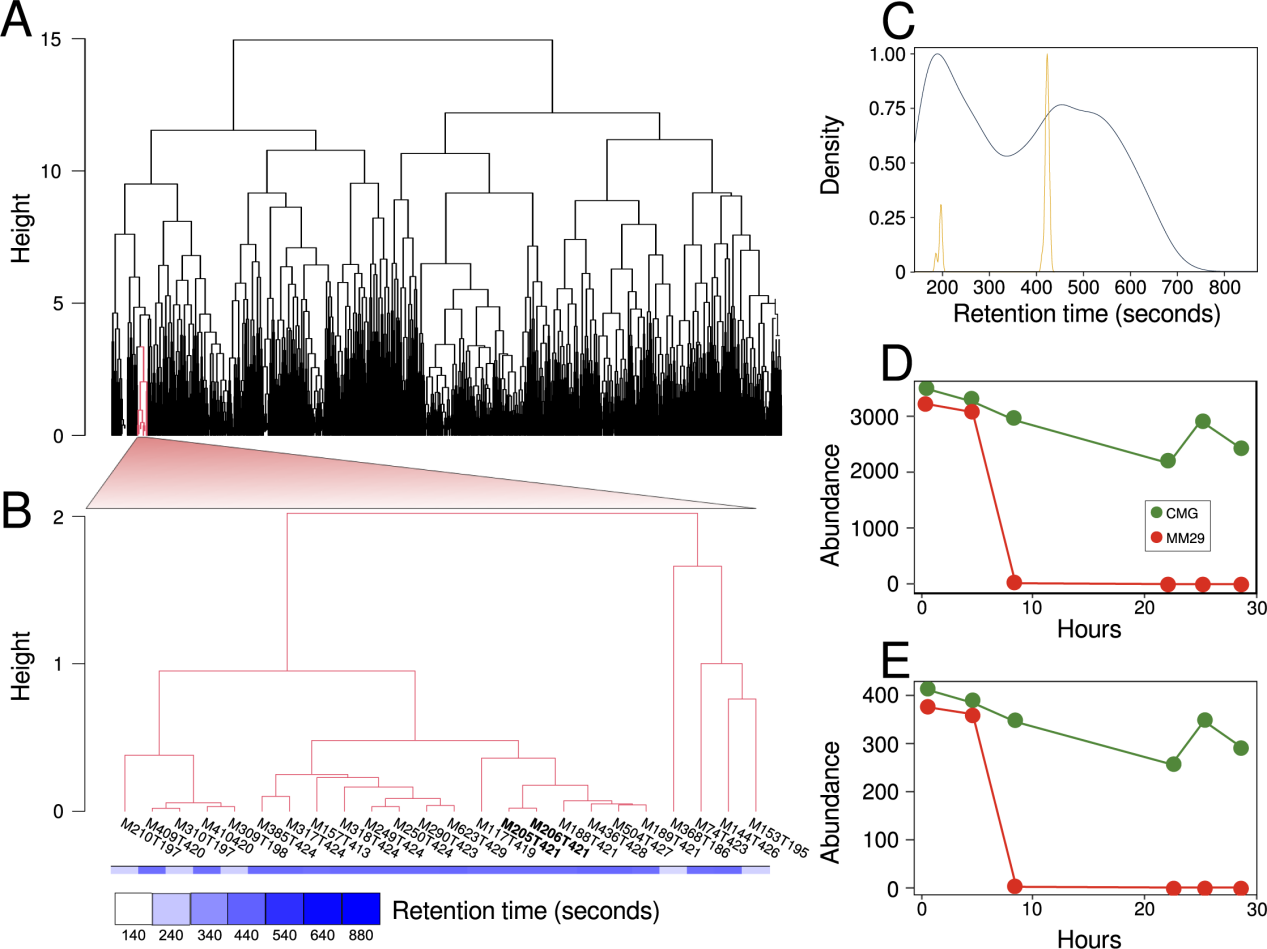
Identification of candidate repressors of ClbP activity in *E. coli* ATCC 25922 cultures using untargeted metabolomics. Metabolite abundances were measured by LC-MS for cultures growing in either MM29 or CMG medium at six-time points (0, 4, 8, 22, 25, and 29 hours) after inoculation. (**A**) Dendrogram showing hierarchical clustering of the abundance profiles for 1,515 metabolite features based on a dissimilarity matrix produced using dynamic time warping. (**B**) Dendrogram of metabolite features in the target cluster showing selective depletion in MM29 relative to CMG medium. Colored bars below dendrograms show retention time. (**A and B**) The height (distance) between cluster members using the complete agglomeration method is shown on the *y*-axis. (**C**) Kernel density estimation shows the distribution of retention times for all 1,515 metabolites (blue) and 24 metabolites in the target cluster (yellow). (**D**) Relative abundance profiles of metabolite spectra for (**D**) M205T421 and (**E**) M206T421 that both have tryptophan as the top hit in the mzCloud database. Metabolite abundances are based on peak areas of extracted ion chromatograms. LC-MS, liquid chromatography–mass spectrometry.

To find candidate metabolites that repress ClbP activity, we performed untargeted metabolomics on a time series of supernatants from ATCC 25922 cultures growing in CMG or MM29 media (Table S1A). We reasoned that the abundance of a metabolite that represses ClbP activity in CMG relative to MM29 medium would be progressively depleted in MM29 medium, but remain replete in CMG. Upon hierarchical clustering of all 1,515 metabolite features across the time series (Fig. 2A), we identified a cluster of 24 metabolites with relative abundance profiles consistent with our proposed profile of a ClbP repressor (Fig. 2B; Fig. S7; Table S1B). Most features in this cluster showed similar retention times near 420 seconds (Fig. 2B and C; Table S1B), suggesting they have comparable hydrophobicities.

To characterize this cluster of metabolite features selectively depleted in MM29 medium, we performed LC-MS/MS on the cluster members (Table S2A) and queried the MS/MS spectra against the mzCloud database to putatively identify seven metabolite features with HighChem HighRes identity scores >50 (Table S2B). We identified two metabolite features, M205T421 and M206T421, for which tryptophan was the top hit in the mzCloud database. The *m*/*z* of their precursor ions is consistent with the monoisotopic mass of protonated tryptophan (M205T421) or a protonated tryptophan isotopologue (M206T421) (Table S2B). M205T421 and M206T421 have similar retention times of 420.33 and 420.20 seconds (Table S1B) and their relative abundance profiles are highly similar showing depletion in MM29 versus CMG medium (Fig. 2D and E).

### Tryptophan reduces ClbP activity and DNA crosslinking

**Fig 3:**
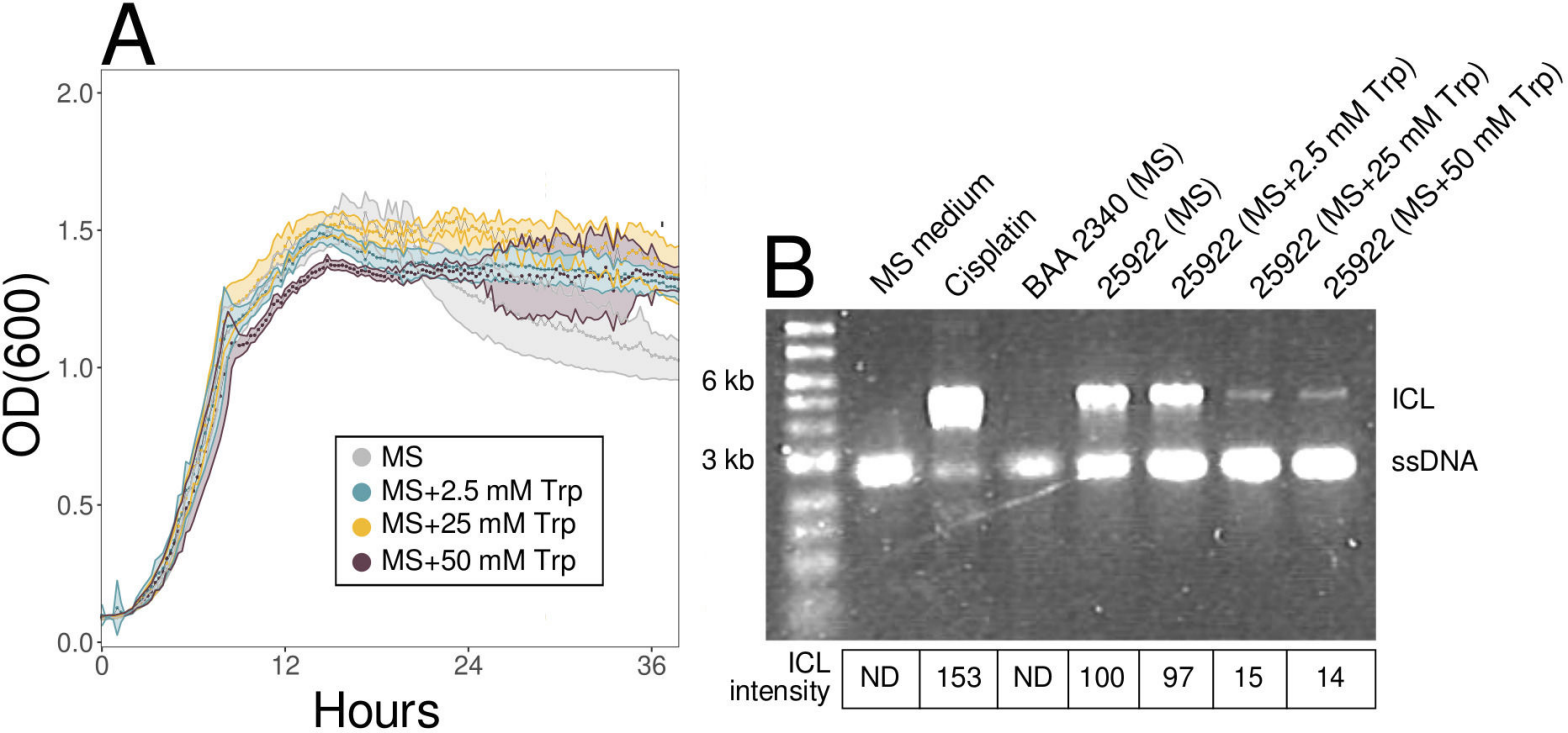
Effect of L-tryptophan supplementation on growth and DNA crosslinking activity of *pks+ E. coli* ATCC 25922 cultures. (**A**) Growth of *E. coli* ATCC 25922 cultures in MS medium (gray) or MS medium supplemented with either 2.5 mM (light blue), 25 mM (yellow), or 50 mM (purple) L-tryptophan. Data points are means of triplicate cultures, shaded regions are ±SD. (**B**) DNA crosslinking assay performed on *Bam*HI-linearized pUC19 DNA that was incubated for 6 hours at 37°C with either MS medium, *E. coli* ATCC BAA 2340 (*pks*−) in MS medium, *E. coli* ATCC 25922 (*pks+*) in MS medium at different Trp concentrations, or 80 µM cisplatin. pUC19 single-stranded DNA is at 2,686 bp; pUC19 ICL DNA is at 5,372 bp. Percent ICL band intensities relative to ATCC 25922 cultures in MS medium shown below gel image. OD_600_, optical density at 600 nm; L-tryptophan, Trp; ICL, interstrand crosslinked; ND, not detected.

We investigated how L-tryptophan supplementation of *E. coli* ATCC 25922 cultures in defined (MS) medium affected growth and ClbP activity. Supplementation of MS medium with up to 50 mM L-tryptophan did not affect growth (Fig. 3A). However, 7H4M fluorescence was 40% reduced by adding 25 mM L-tryptophan to MS medium without affecting pH (Fig. S8), complicating the use of ClbP-17 to measure the effect of L-tryptophan on ClbP activity. More generally, the sensitivity of 7H4M fluorescence to pH (Fig. S4), L-tryptophan (Fig. S8), and likely other metabolites show the challenges of using the ClbP-17 activity-based probe to investigate ClbP activity.

As an alternative assay to ClbP-17 to assay colibactin, we tested if L-tryptophan supplementation reduced *in vitro* cross-linking (ICL) of DNA. DNA damage by colibactin results from intercalation by the thiazoline-thiazole tail and subsequent alkylation by the electrophilic cyclopropane, resulting in covalent crosslinking of complementary DNA strands (15, 16, 29). As ICL prevents the denaturation of DNA strands, it can be observed as an apparent doubling of the molecular weight of linearized plasmid DNA on a denaturing agarose gel (29). As expected, treatment with the drug cisplatin resulted in nearly complete ICL (Fig. 3B; Fig. S9A). DNA crosslinking occurred with *E. coli* ATCC 25922 (*pks+*), but not *E. coli* ATCC BAA 2340 (*pks*−), when grown in MS medium (Fig. 3B; Fig. S9A). Quantification of band intensities showed that relative to MS medium alone, supplementation of *E. coli* ATCC 25922 cultures with 25 mM or 50 mM L-tryptophan reduced ICL by 85% and 86%, respectively (Fig. 3B; Fig. S9A). These results support that supplementation of *pks+ E.coli* cultures with L-tryptophan reduces colibactin-mediated DNA damage.

### Tryptophan elevates gene expression for ammonium assimilation and copper export

**Fig 4:**
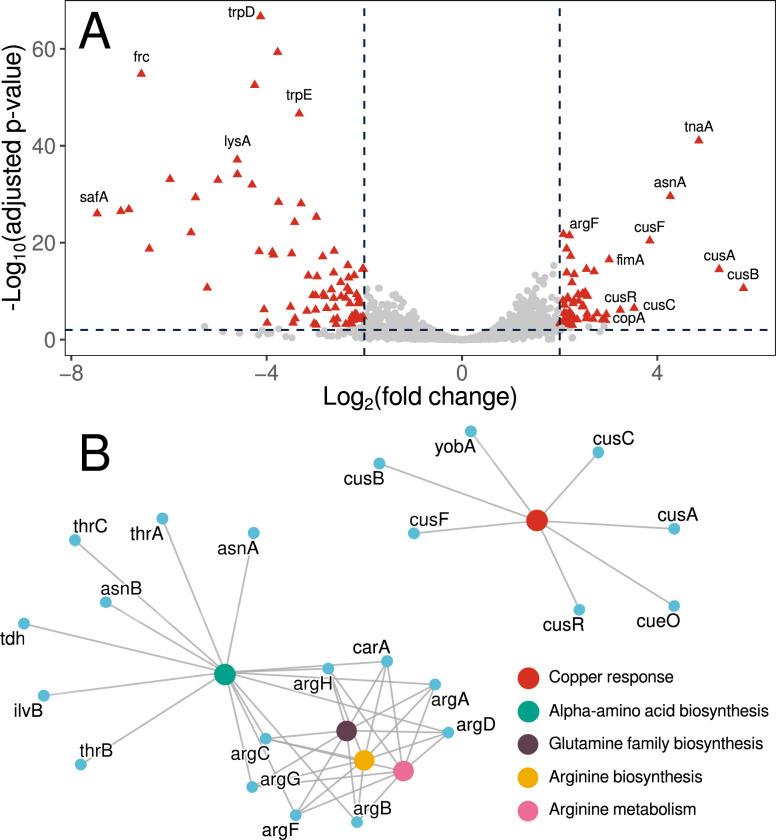
Gene expression of *E. coli* ATCC 25922 cultures growing in MS medium with and without 25 mM L-tryptophan supplementation measured by RNA-seq. (**A**) Volcano plot of mRNA expression in L-tryptophan supplemented cultures relative to MS medium. The 137 differentially-expressed [>2 log_2_(fold change), *P*-value < 0.001] genes are shown as red triangles; all other genes are gray circles. Names of selected genes are shown. (**B**) Gene networks showing Gene Ontology linkages of functional pathways whose expression increased upon L-tryptophan supplementation (*P*-value < 0.01). The *P*-values were corrected for multiple hypothesis testing using the Benjamini and Hochberg method.

To examine the mechanism by which L-tryptophan may reduce colibactin activity, we quantified genome-wide mRNA expression differences between log phase *E. coli* ATCC 25952 cultures growing in MS medium with 25 mM L-tryptophan supplementation (+Trp) versus MS medium alone (−Trp) (Table S3). As expected, *tnaA*, encoding the tryptophanase that splits tryptophan into indole, pyruvate, and ammonia, and the *tnaB* tryptophan permease were highly upregulated in the +Trp treatment (Fig. 4A). To assimilate the liberated ammonia, genes were upregulated to synthesize carbamoyl phosphate (*carA*) and ornithine (*argABCD*) and to incorporate these metabolites into arginine (*argFGH*) (Fig. 4A and B). Also as expected, the *trp* genes for tryptophan biosynthesis were highly repressed in the +Trp treatment (Fig. 4A). However, *clbP* was not differentially expressed in response to L-tryptophan and *clbI* was the only gene among 19 genes in the *clb* island (*clbA-clbS*) that was repressed (adjusted *P*-value < 0.01, Table S3). Thus, reduced colibactin activity upon L-tryptophan supplementation is unlikely to be the result of transcriptional repression of the *clb* genes.

Genes encoding all three systems to mitigate copper toxicity in *E. coli* (30) were highly upregulated in response to L-tryptophan. The *cus* genes (*cusCBA, cusF*, and *cusR*) and *copA* were among the 10 most highly upregulated genes in the +Trp treatment; *cueO* was also upregulated to a lesser extent (Fig. 4B; Table S3). The CusCBA efflux system is an RND-type efflux system that transports copper ions from the cytosol to the periplasm and outside of the cell (31), CopA is a P-type ATPase that transports cytoplasmic copper to the periplasm (32), and CueO oxidizes Cu(I) to the less toxic Cu(II) (33).

### Tryptophan and copper interact to alter growth, colibactin activity

**Fig 5:**
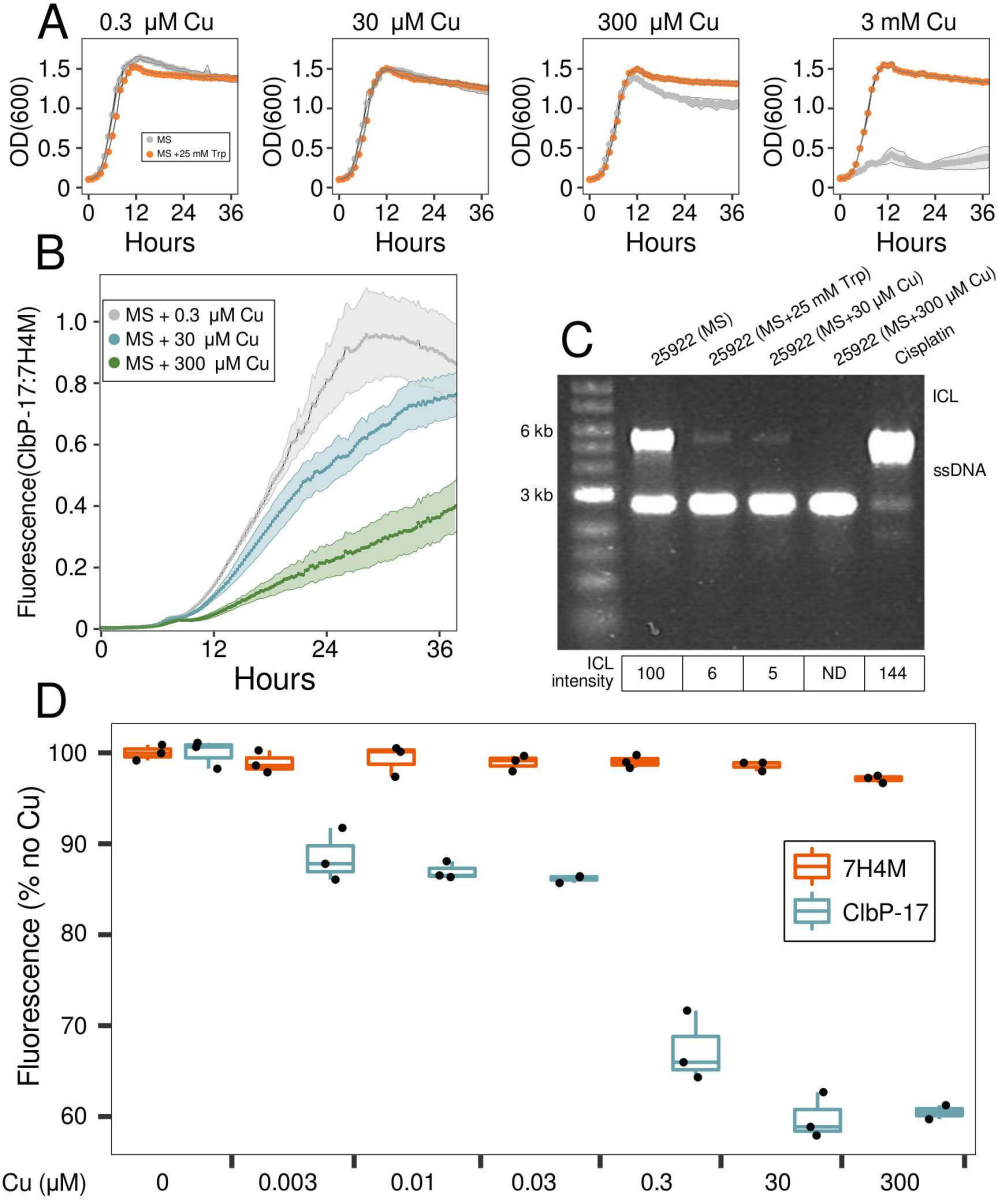
Effect of L-tryptophan and copper supplementation on *E. coli* ATCC 25922 growth, ClbP activity, and DNA cross-linking. (**A**) Growth of cultures in MS medium (gray) or in MS medium with 25 mM tryptophan (orange) at different copper concentrations (shown above plots). Data points are means of triplicate cultures, shaded areas are ±SD. (**B**) ClbP-17:7H4M fluorescence of cultures growing in MS medium containing either 0.3 µM (gray), 30 µM (blue), or 300 µM (green) copper chloride. Fluorescence of ClbP-17 and 7H4M was measured as 360 ± 40 nm excitation, and 440 ± 20 nm emission in triplicate cultures. ClbP-17:7H4M is the fluorescence of ClbP-17 divided by the median 7H4M fluorescence. (**C**) DNA crosslinking assay performed on *BamH*I-linearized pUC19 DNA that was incubated for 6 hours at 37°C with 80 µM cisplatin or with cultures of *E. coli* ATCC 25922 in MS medium (0.3 µM Cu) or MS medium supplemented with either 25 mM Trp or Cu (30 µM, 300 μM). pUC19 single-stranded DNA is at 2,686 bp; pUC19 ICL DNA is at 5,372 bp. ICL band intensities relative to ATCC 25922 cultures in MS medium are shown below the gel image. (**D**) Activity of purified ClbP peptidase domain (ClbP-pep) in buffer containing different Cu concentrations. ClbP-17 fluorescence (blue) is shown as a percentage of the mean fluorescence in the absence of copper and is compared to 7H4M fluorescence (red) at the same Cu concentrations. Box plots show median and interquartile ranges; data points are individual measurements. (**B, D**) ClbP-17 and 7H4M fluorescence were measured at 360 ± 40 nm excitation, and 440 ± 20 nm emission. OD_600_, optical density at 600 nm; SD, standard deviation; Cu, copper; Trp, L-tryptophan; ICL, interstrand crosslinked DNA; ND, not detected.

To investigate connections between L-tryptophan and copper export, we measured the growth of ATCC 25922 cultures at different copper chloride concentrations in MS medium (−Trp) and in MS medium with 25 mM L-tryptophan (+Trp). Growth of −Trp cultures was slightly reduced by 300 µM copper (maximum cell density 14% lower relative to 0.3 µM copper) and strongly inhibited by 3 mM copper (maximum cell densities 75% lower relative to 0.3 µM copper), whereas growth in +Trp cultures was not affected by these copper concentrations (Fig. 5A). L-tryptophan thus protects *E. coli* ATCC 25922 from copper toxicity, likely by sequestering copper and reducing its intracellular accumulation.

As L-tryptophan stimulated the expression of the gene to efflux copper from the cell, we examined the effect of adding copper to the media on ClbP-17 fluorescence. In contrast to L-tryptophan, increased copper concentration had little effect on 7H4M fluorescence. Fluorescence of 7H4M was only 4% lower when the copper concentration in the MS medium was increased from 0.3 μM to 300 µM (Fig. S8B), supporting that ClbP-17 can be used to assess the effect of copper on ClbP activity. We observed a reduction in ClbP-17:7H4M fluorescence in ATCC 25922 cultures when copper was increased from 0.3 μM to 30 µM or 300 µM (Fig. 5B). Similarly, copper supplementation inhibited ICL of plasmid DNA by ATCC 25922 (Fig. 5C; Fig. S9B). Quantification of band intensities showed that relative to MS medium alone, ICL of plasmid DNA was reduced by supplementation with either 25 mM tryptophan, 30 µM copper, or 300 µM copper by 94%, 95%, or >99%, respectively (Fig. 5C). Reduction of ClbP-17 fluorescence and ICL activity by copper in *E. coli* ATCC 25922 cultures was particularly interesting as copper has previously been shown to increase both the degradation and the *in vitro* DSB activity of a purified colibactin metabolite (34).

Based on copper-reducing ClbP-17:7H4M fluorescence and ICL of extracellular DNA in ATCC 25922 cultures, we hypothesized that copper may directly inhibit the peptidase function of ClbP. We purified the ClbP peptidase domain (ClbP-pep) using a C-terminal His tag and confirmed its ability to cleave ClbP-17 (Fig. S10). We then measured the effect of increasing copper concentrations on ClbP-17 cleavage by ClbP-pep. ClbP-17 cleavage by ClbP-pep is progressively inhibited by increasing copper concentrations, whereas 7H4M fluorescence is unaffected (Fig. 5D). While the activity profile of ClbP-pep has been found to be different from full-length ClbP (14), our results suggest that the reduction in ClbP activity and ICL in ATCC 25922 cultures supplemented with copper (Fig. 5B and C) is associated with a direct effect of copper inhibiting ClbP-pep (Fig. 5D). Together, these observations suggest that copper export to the periplasm could be responsible for the L-tryptophan-mediated reductions in ClbP activity and DNA damage.

## DISCUSSION

Colibactin-encoding *E. coli* cells are present in 60% of humans with familial adenomatous polyposis or CRC and the colibactin mutational signature is present in human cancer patient cells (10, 11), highlighting the importance of developing strategies to reduce colibactin activity. In this study, we identified metabolites that repress ClbP activity (ClbP-17:7H4M fluorescence) and the DNA crosslinking activity (ICL) of colibactin in *pks+ E. coli*. Untargeted metabolomics revealed L-tryptophan as a potential ClbP repressor, which was supported by supplementing cultures with L-tryptophan to reduce extracellular DNA crosslinking (Fig. 3B). L-tryptophan supplementation did not affect *clb* gene expression, but stimulated the expression of copper export genes (Fig. 4A) and protected from copper toxicity (Fig. 5A). Increasing copper in ATCC 25922 cultures reduced ClbP-17:7H4M fluorescence (Fig. 5B) and extracellular DNA crosslinking (Fig. 5C), and inhibited the *in vitro* activity of purified ClbP-pep (Fig. 5D).

Similar to ClbP, copper inhibits the activities of other proteases such as *Helicobacter pylori* HtrA (35), the 20S proteasome (36), and the protease from human immunodeficiency virus 1 (37). Copper-mediated inhibition of ClbP could be a mechanism to regulate colibactin production to protect *pks*+ cells from autotoxicity, similar to ClbS that hydrolyses the cyclopropane warhead of colibactin (38, 39). In a previous study, copper enhanced both the instability and the activity of a colibactin metabolite that acts by a copper-mediated oxidative mechanism to induce DNA double-strand breaks (34). Combined with our data that supplementation of ATCC 25922 cultures with copper reduces DNA crosslinking (Fig. 5C), it appears that copper has a dual role to both limit and enhance the DNA-damaging activity of colibactin metabolites.

**Fig 6:**
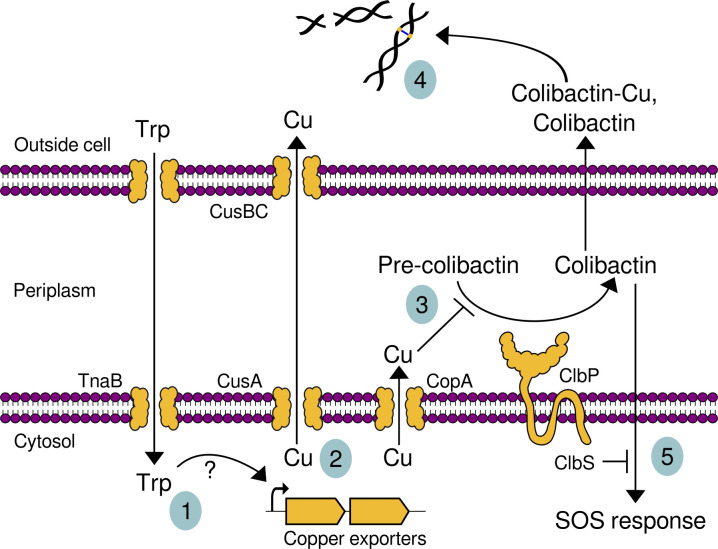
Model for how tryptophan reduces DNA damage by colibactin through copper-mediated repression of ClbP activity. (1) Uptake of tryptophan stimulates transcription of copper exporters by an unknown mechanism. (2) Copper exporters pump copper to the periplasm and outside cells. (3) Periplasmic copper inhibits ClbP activity, reducing the processing of precolibactin to active colibactin. (4) Colibactin and colibactin-copper complexes cause DNA crosslinking and double-strand breaks. (5) ClbS protects the colibactin-producing cell from DNA damage by colibactin, which otherwise activates the SOS response.

We integrated our data into a model of how L-tryptophan and copper interact to repress ClbP activity and DNA crosslinking by *pks+ E. coli* (Fig. 6). In this model, (i) L-tryptophan uptake stimulates the expression of copper exporters. While the mechanism by which L-tryptophan supplementation stimulates the expression of copper exporters is unknown, cellular amino acids sequester copper (40) and, in particular, tryptophan complexes with copper (41). The uptake of tryptophan-copper complexes could lead to intracellular copper accumulation and consequent induction of the copper export genes. Furthermore, the protective effect of L-tryptophan from copper toxicity suggests that either tryptophan increases the efficiency of copper export or that intracellular tryptophan mitigates copper toxicity. (ii) Expression of copper exporters pump copper into the periplasm and extracellular milieu. (iii) Copper in the periplasm could inhibit ClbP, resulting in lower processing of precolibactin into mature colibactin. (iv) Colibactin, perhaps complexed with copper, mediates DNA damage outside the cell. (v) DNA damage by colibactin in the producing cells is mitigated by ClbS.

Elucidating mechanisms regulating colibactin activation and genotoxicity can lead to novel strategies to repress its activity in the gut and ultimately prevent colibactin-mediated carcinogenesis. Pathogenicity of *Enterobacteriaceae* is typically controlled using antibiotics such as aminoglycosides, fluoroquinolones, and B-lactams. However, antibiotics can have negative health consequences by altering the gut microbiome and leading to the emergence of multi-drug resistant strains (42). Studies have identified small molecule inhibitors of the genotoxic activity of colibactin-producing *E. coli* (43, 44). Repression of colibactin activity using L-tryptophan is particularly attractive due to its other beneficial effects such as prevention of *E. coli* biofilm formation (45). In addition, tryptophan metabolites from the gut microbiota stimulate aryl hydrocarbon receptor-mediated IL-22 signaling to reduce mucosal inflammation and protect from *Candida albicans* colonization (46).

Additional research is needed to assess the efficacy of using molecules such as L-tryptophan or copper to repress colibactin production and activity in humans. In this study, we focused on strain ATCC 25922 as a model *pks+ E. coli*, but humans harbor various *pks*+ strains that can differ in their metabolic and genetic regulatory features. Moreover, studies are needed to show that metabolite concentrations needed to repress colibactin activity in *pks+ E. coli* can be achieved in the gut. Previous studies have found that 4%–6% of tryptophan is metabolized to indoles by the gut microbiota (47) and that fecal indole concentrations in healthy adults were 2.59 mM (48). Intestinal copper concentrations have been estimated to reach 10 µM (49). Thus, the tryptophan and copper concentrations used in this culture-based study may be achievable in the human intestine. Other studies have identified other metabolites such as D-serine, cinnamaldehyde, and medicinal plant extracts that reduce *E. coli clbB* expression and colibactin cytotoxicity (20, 50, 51). Our results complement these studies to advance the development of potent, antibiotic-sparing prophylactics that inhibit colibactin genotoxicity and its tumorigenic effects.

## MATERIALS AND METHODS

### Microbial growth

Cultures of *E. coli* ATCC 25922 (*pks+*) or BAA 2340 (*pks*−) were grown at 37°C in either MS medium (52) (Table S4), MM29 medium (26), or CMG medium (Anaerobe Systems, CA, USA). Cultures growing in MS medium were supplemented with L-tryptophan (Sigma T0254) or copper chloride dihydrate (CuCl_2_·2H_2_O) (Sigma 307483), as appropriate. Growth (optical density at 600 nm [OD_600_]) and all fluorescence measurements were made using a CLARIOstar microplate reader (BMG Labtech, Ortenberg, Germany).

### BCECF method for pH measurements

The pH of cultures was measured using the BCECF method (25, 26). BCECF (C3411 Sigma) at 2 µM was added to cultures and fluorescence at the pH-sensitive point (485 nm excitation; 540 nm emission) relative to the pH-insensitive isosbestic point (450 nm excitation; 540 nm emission) was calculated. Culture pH was computed using a four-parameter log-logistic (4PL) regression that was fit to BCECF fluorescence for each growth medium at experimentally adjusted pH 4–9 (Fig. S5). The 4PL regression was calculated using the R package drc version 3.0-1.

### ClbP activity-based probe (ClbP-17)

ClbP-17 (21) was synthesized from N-Boc-D-asparagine using a five-step process yielding the product at 98% purity (WuXi AppTech, Shanghai China) (Fig. S1). Ten mM (100×) stocks of ClbP-17 and 7H4M (Fischer 10377230) were prepared in DMSO and stored at −20°C. ClbP-17 and 7H4M were added to cultures or ClbP-pep at a final concentration of 100 µM. ClbP-17 and 7H4M fluorescence (360 ± 40 nm excitation, 440 ± 20 nm emission) were measured using a CLARIOstar microplate reader (BMG Labtech, Ortenberg, Germany). The ClbP-17:7H4M ratio was calculated for triplicate cultures containing each molecule as the ratio of ClbP-17 fluorescence to the median 7H4M fluorescence.

### Liquid chromatography–mass spectrometry

Culture supernatant samples for metabolomics were taken 0, 4, 8, 22, 25, and 29 hours post inoculation by centrifuging cultures at 3000 × *g* for 5 minutes, removing the supernatant, and freezing at −80°C. Metabolite analysis was performed by liquid chromatography (Thermo Dionex Ultimate 3000) coupled with a Thermo Scientific Q Exactive mass spectrometer. Full MS analysis was performed in positive mode using *m*/*z* range from 50 to 750 *m*/*z* at a resolution of 35,000 with the following source parameters: spray voltage 4,200 V, capillary temperature 320°C, sheath gas at 40 units, auxiliary gas at 10 units, and S-lens radio frequency level at 50%. Samples were injected onto a Waters Acquity UPLC BEH amide column (2.1 mm × 150 mm, 1.7 µm, 130 Å) with a 60°C column temperature. Mobile phase A was 2.5 mM ammonium formate with 0.1% formic acid in 95:5 acetonitrile:water. Mobile phase B was 2.5 mM ammonium formate with 0.1% formic acid in 5:95 acetonitrile:water. Water, ammonium formate, and formic acid were LC-MS grade (Thermo Fisher, Waltham, MA). Mobile phase B gradient was as follows: 0 minutes: 10%, 2 minutes: 10%, 12 minutes: 60%, 13 minutes: 80%, 15 minutes: 80%. A 2-minute ramp back to 10% and 3-minute equilibration was performed between each sample. The volume of each injection was 4 µL.

To prepare samples for LC-MS, an internal standard (IS) solution containing 2 µM leucine-d10 (Sigma-Aldrich, St Louis, MO) and 2 µM indole-3-acetic acid-d7 (Cambridge Isotope Laboratories, Tewksbury, MA) was prepared in methanol and chilled. An aliquot of each culture sample was combined into a pooled sample, which was treated using the same methods as each culture sample. Each culture sample (30 µL) was mixed with 170 µL of methanol IS solution, vortexed, and centrifuged at 1,800 × *g* for 10 minutes at 4*°*C. The supernatant (150 µL) was extracted and dried in a speed vacuum for 2 hours without light or heat. Samples were resuspended in 100 µL of 50:50 methanol:water, vortexed, and centrifuged at 1,800 × *g* for 10 minutes at 4*°*C. An aliquot of each sample (10 µL) was combined into a pooled sample. The remaining sample (80 µL) was extracted and transferred to an LC injection vial.

Full MS data were processed to generate abundance profiles based on MS1-extracted ion chromatograms using XCMS (53). Peak parameters and alignment were determined and processed to achieve good correspondence using the IS solution and multiply injected pooled samples. Results were trimmed against the pooled QC to remove noisy and uninformative features, then Pareto scaled. Metabolite features were clustered based on their abundance profiles across the six-time points in MM29 and CMG media. The 12 abundance values for each metabolite (six-time points, two media) were scaled (per feature) to generate a distance matrix using the dynamic time warping algorithm implemented in the TSClust R package (version 1.3.1) (54). Hierarchical clustering was performed on the distance matrix of metabolite features using the complete agglomeration method as implemented in the hclust function from the stats R package (version 3.1.0). Manually searching the metabolite features identified a target cluster having relative abundance profiles matching a potential colibactin repressor.

The chemical structures of features in the target cluster were investigated by re-injecting the pooled sample and performing LC-MS/MS with parallel reaction monitoring at a resolution of 17,500, AGC target 2e5, and maximum IT 100 ms. Higher energy C-trap dissociation was collected with normalized collision energy (NCE) 30 and NCE 90. Source and LC conditions were the same as for MS1 data. MS1 chromatogram peaks corresponding with the retention time and mass of the target features were identified using Xcalibur version 3.1 (Thermo Fisher). The corresponding MS2 spectrum was then extracted and queried against the mzCloud database (Table S2B).

### DNA crosslinking assay

The colibactin activity of *E. coli* cultures was tested using a DNA crosslinking assay based on denaturing gel electrophoresis under alkaline conditions to enable migration of single-stranded DNA (29). *E. coli* BAA 2340 (*pks*−) was cultured in MS medium and *E. coli* ATCC 25922 (*pks+*) was cultured in MS medium ± L-tryptophan or copper supplementation, as appropriate. Cultures of 200 µL were grown for 6 hours at 37°C, spiked with 1 µg of *Bam*HI-linearized pUC19 plasmid DNA, and incubated for an additional hour. Cisplatin-treated controls were prepared by incubating 1 µg of *BamH*I linearized pUC19 DNA for 6 hours at 37°C with 80 µM cisplatin in 1× phosphate-buffered saline.

Plasmid DNA from culture supernatants and cisplatin treatments was purified using a Qiagen QIAquick PCR kit (Qiagen, Germany) and resolved by denaturing gel electrophoresis. Briefly, 1% agarose gels were prepared in a 100 mM sodium chloride, 2 mM EDTA (pH 8) solution and soaked in an electrophoresis running buffer (40 mM NaOH, 1 mM EDTA). DNA (200 ng per lane) was resolved by electrophoresis for 45 minutes at 1 V cm^−1^ and then for 2 hours at 2 V cm^−1^. The gel was soaked in a neutralization solution (150 mM sodium chloride, 100 mM Tris pH 7.4) for 4 hours with the buffer changed after each hour, DNA was stained with Gel Red (Biotium, Fremont, CA), and visualized. Gel band intensities were compared using ImageJ software version 1.54d (55).

### RNA sequencing

Cultures of *E. coli* ATCC 25922 were grown in MS medium with and without supplementation with 25 mM L-tryptophan to mid-log phase. RNA was extracted using TRI reagent (Sigma 93289) (56) and 1 µg of total RNA was depleted of rRNA by the NEBNext rRNA depletion kit (New England Biolabs, MA, USA). RNA was purified using NEBNext RNA sample purification beads (New England Biolabs, MA, USA) into 5 µL of water. Quantity and quality of rRNA-depleted RNA were assessed on a Qubit 2.0 Fluorometer (Invitrogen, Carlsbad, CA, USA) and an Agilent 2100 Bioanalyzer (Agilent Technologies, USA). cDNA libraries were prepared using the NEBNext Ultra II directional RNA library preparation kit (New England Biolabs, MA, USA). RNA fragmentation was performed for 90 seconds at 94°C and incubated for 60 minutes at 42°C for first-strand cDNA synthesis. NEBNext adaptors were diluted five times for adaptor ligation and 16 PCR cycles were applied for enrichment of adaptor-ligated DNA. After profile analysis by a Bioanalyzer and qPCR quantification on MxPro equipment (Stratagene, La Jolla, USA), libraries were sequenced on an Illumina Novaseq 6000 under a PE-50 mode.

Sequencing reads were trimmed of low-quality nucleotides (Q < 20) from both ends of the reads, sequencing adaptors, and primer sequences. Sequences between the second unknown nucleotide and the end of the read were removed and read shorter than 30 nucleotides (after trimming) were discarded. RNA sequencing reads were mapped to the *E. coli* ATCC 25922 genome (NCBI Genbank accession CP009072) using Bowtie 2 (57). Reads per genome feature were counted from BAM files using featureCounts (58) and differentially expressed genes were identified using DEseq2 (59). Gene Ontology terms were used to calculate functional pathway enrichments (*P* < 0.01) using clusterProfiler 4.0 (60).

### ClbP purification and activity measurements

The peptidase domain of ClbP (ClbP-pep), as defined in (13), was PCR amplified with a C-terminal His tag using primers clbP_F (5-AAAGAAGGAGATAGGATCATGACAATAATGGAACACGTTAG-3) and clbP_R (5-GTGTAATGGATAGTGATCTTAATGGTGATGGTGATGATGATATTTGCCAATGCGCAGA-3). The PCR product was cloned by ligation-independent cloning into pET-22B(+) (61, 62) and the forward and reverse sequences were confirmed by sequencing. Plasmids were transformed into *E. coli* BL21(DE3) (Novagen 70235). A culture of *E. coli* BL21(DE3) expressing ClbP-pep was grown in 100 mL TB medium to OD_600_ = 2, induced by adding 500 µM IPTG, and incubated overnight at 20°C. Cells were pelleted and resuspended in lysis buffer (50 mM phosphate buffer pH   8, 0.5 M NaCl, 30 mM imidazole, 15% glycerol, 1 mM pefabloc [Sigma 76307]). Cells were lysed by sonication (Cole-Parmer Vibracell CV33) in the presence of 1 mg mL^−1^ lysozyme (Novagen 71230). ClbP-pep was purified using a Ni-NTA spin column (Qiagen 31014) and quantified by Bradford assay. Purified ClbP-pep was visualized on 12% SDS-PAGE gels (Novex 12% Bis-Tris Gel NP0342BOX) (Fig. S10).

The activity of ClbP-pep was measured by diluting the enzyme to 0.01 µg µL^−1^ in buffer (50 mM Tris, 200 mM sodium chloride, pH 8) containing 100 µM of either ClbP-17 or 7-hydroxy-4-methylcoumarin (Fisher Scientific AC156380250) and copper chloride dihydrate (Sigma 307483), as appropriate. ClbP-pep was then incubated for 120 minutes at 37°C and fluorescence was measured (360 ± 40 nm excitation, 440 ± 20 nm emission) using a CLARIOstar microplate reader (BMG Labtech, Ortenberg, Germany).

## Data Availability

The RAW files for mass spectrometry analysis were deposited in the MassIVE database (accession MSV000091164). FASTQ files for RNA sequencing were deposited in the European Nucleotide Archive (accession ERP163145).

## SUPPLEMENTAL MATERIAL

Supplemental Figures msystems.00992-24-s0001.pdf Figures S1-S10.

Table S1 msystems.00992-24-s0002.xlsx Metabolite abundances.

Table S2 msystems.00992-24-s0003.xlsx Chromatogram and mass spectrum data.

Table S3 msystems.00992-24-s0004.xlsx Differential mRNA expression.

Table S4 msystems.00992-24-s0005.xlsx MS medium recipe.

ASM does not own the copyrights to Supplemental Material that may be linked to, or accessed through, an article. The authors have granted ASM a non-exclusive, world-wide license to publish the Supplemental Material files. Please contact the corresponding author directly for reuse.
