# Supplementary material for: L-tryptophan and copper interactions linked to reduced colibactin genotoxicity in pks+ Escherichia coli
Source: mSystems. 2024 Sep 12;9(10):e00992-24. doi: 10.1128/msystems.00992-24 (PMC11495049; doi:10.1128/msystems.00992-24)
Supplement: Supplemental Figures — Figures S1-S10. [file msystems.00992-24-s0001.pdf]

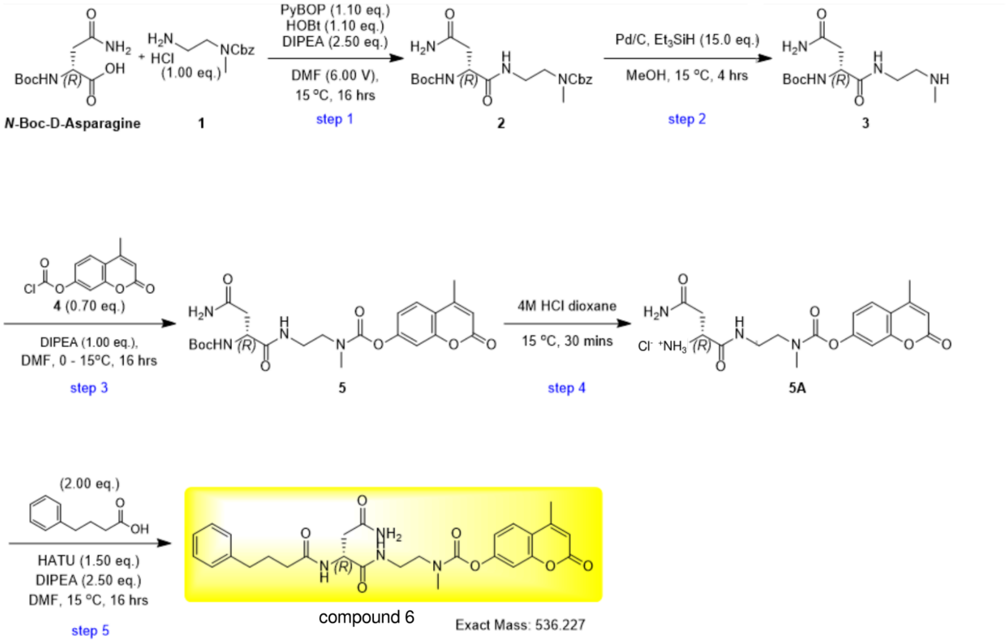

Fig S1 Five step synthesis scheme of the ClbP activity-based probe ClbP-17 starting from N-Boc-D-asparagine.

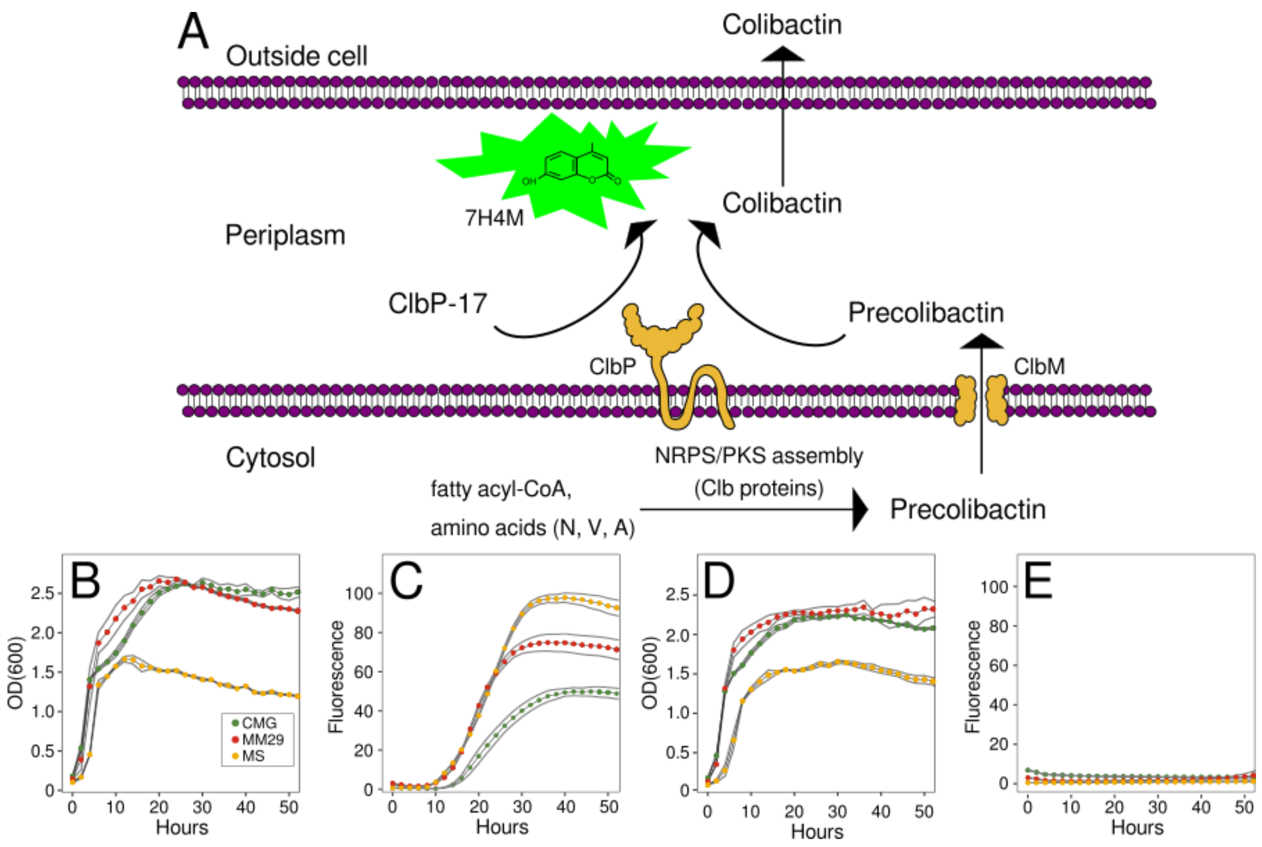

Fig S2 (A) Cell diagram of colibactin synthesis and measurement of ClbP activity using activity-based probe ClbP-17. (B-E) Growth and ClbP-17 fluorescence of ±*clb E. coli* strains in different media. Cultures of (B,C) *pks+ E. coli* ATCC 25922 or (D,E) *pks- E. coli* ATCC BAA 2340 were measured for (B,D) Growth and (C,E) ClbP-17 fluorescence activity in CMG (green), MM29 (red), or MS (yellow) medium containing 100 μM ClbP-17. Growth was measured as optical density at 600 nm (OD_{600}) and fluorescence was measured as 360±40 nm excitation, 440±20 nm emission. Data points are means of triplicate cultures, shaded areas are ±standard deviation. N asparagine, V valine, A alanine, NRPS non-ribosomal peptide synthetase, PKS polyketide synthase.

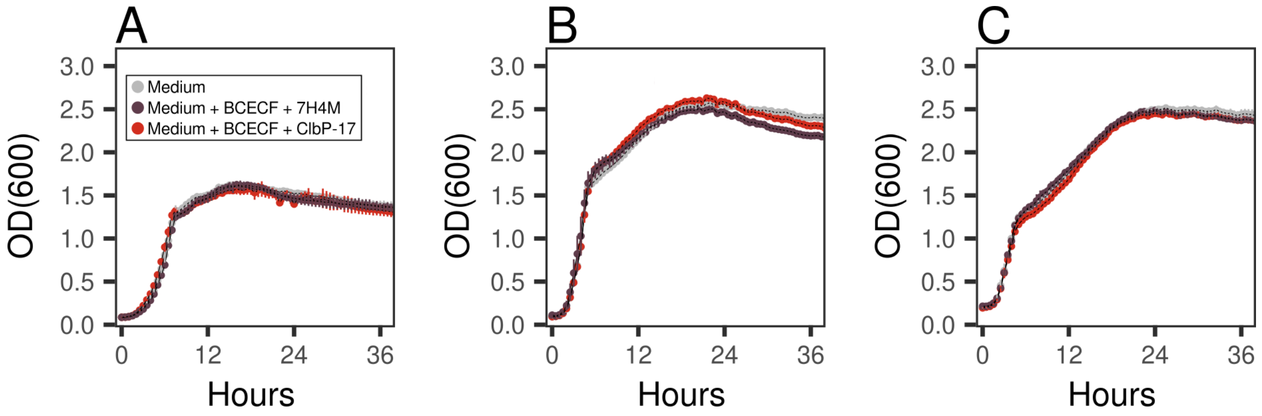

Fig S3 Growth of *E. coli* ATCC 25922 cultures in (A) MS, (B) MM29, or (C) CMG medium with or without addition of BCECF, 7H4M, or ClbP-17. Growth was measured as optical density at 600 nm (OD_{600}) in medium alone (gray), medium supplemented with 2 μM BCECF and 100 μM 7H4M (purple), or medium supplemented with 2 μM BCECF and 100 μM ClbP-17 (red). Data points are means of triplicate cultures, error bars are ±standard deviation and are smaller than symbols where not apparent. BCECF [2,7-bis-(2-carboxyethyl)-5-(and-6)-carboxyfluorescein],7H4M 7-Hydroxy-4-Methylcoumarin.

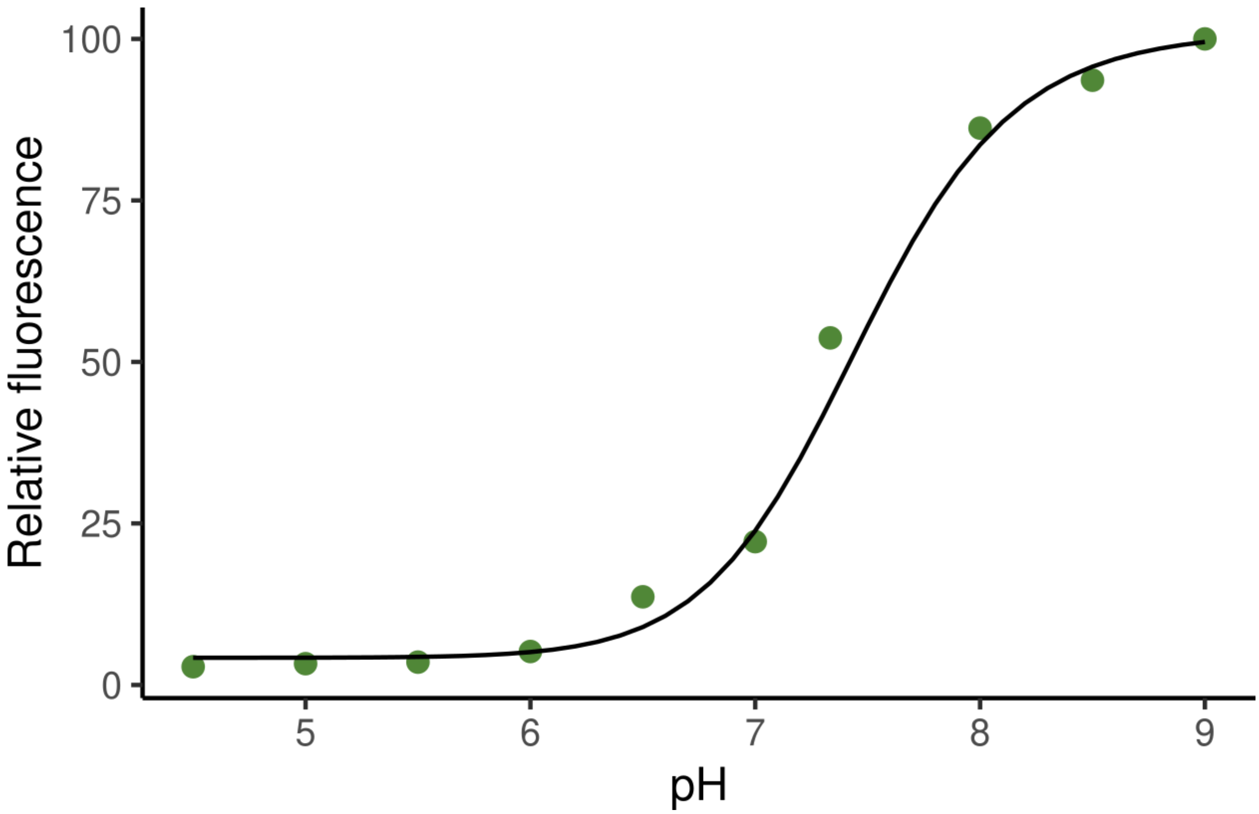

Fig S4 Fluorescence of 7H4M at different pH values. A solution of 100 μM 7H4M was prepared in buffer (50 mM Tris, 200 mM sodium chloride) adjusted to different pH and fluorescence (360±40 nm excitation, 440±20 nm emission) was measured using a CLARIOstar microplate reader (BMG Labtech, Ortenberg, Germany). Plot shows relative fluorescence as a percentage of the maximum. Data points are means of triplicate measurements. The curve shows a four-parameter log-logistic function fit to the data using R package drc version 3.0-1. 7H4M 7-Hydroxy-4-Methylcoumarin.

A

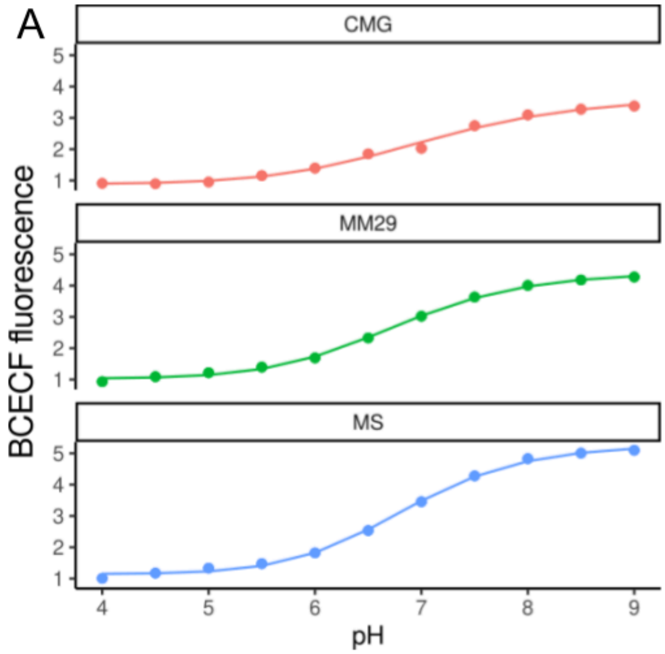

B

$$BCECF\ Fluorescence = d + \frac{a-d}{1+e^{b(log(pH)-log(c))}}$$

a = Minimum Fluorescence (Min)
b = Hill Slope (Hill)
c = Half Maximal Effective Fluorescence (EC50)
d = Maximum Fluorescence (Max)

C

term	medium	estimate	std.error
Hill	MS	-12.25	0.46
Hill	MM29	-11.06	0.52
Hill	CMG	-9.55	0.63
Min	MS	1.14	0.03
Min	MM29	1.03	0.03
Min	CMG	0.89	0.04
Max	MS	5.29	0.05
Max	MM29	4.44	0.05
Max	CMG	3.67	0.08
EC50	MS	6.85	0.02
EC50	MM29	6.78	0.03
EC50	CMG	7.06	0.05

Fig S5 Effect of pH on BCECF fluorescence in growth media. Each growth medium (CMG, MM29, MS) was adjusted to different pH between pH 4-9. BCECF (2 μM) was added to the media and BCECF fluorescence was measured at the pH-sensitive point (485 nm excitation; 540 nm emission) and the pH-insensitive isosbestic point (450 nm excitation; 540 nm emission). (A) BCECF fluorescence is shown as the ratio at the pH-sensitive point to the isosbestic point (485:450). (B) A four-parameter log-logistic function was fit to the curves in each medium using R package drc version 3.0-1. (C) the four parameters from the log-logistic function for each growth medium.

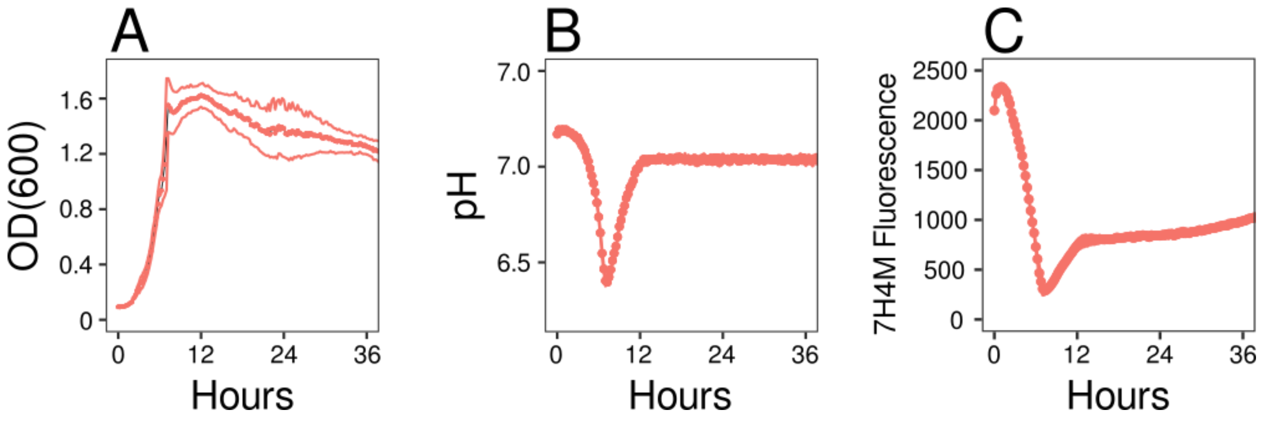

Fig S6 The (A) growth, (B) pH, and (C) 7H4M fluorescence of *E. coli* ATCC 25922 cultures growing in MS medium supplemented with 2 µM BCECF and 100 µM 7H4M. Growth was measured as optical density at 600 nm (OD_{600}). The pH was measured as the ratio of the fluorescence of BCECF at the pH-sensitive point (485±15 nm excitation; 540±20 nm emission) relative to the pH-insensitive isosbestic point (450±15 nm excitation; 540±20 nm emission). 7H4M fluorescence was measured as 360±40 nm excitation, 440±20 nm emission. Data points are means of triplicate cultures, error ranges show ±standard deviation. Measurements were performed using a CLARIOstar microplate reader (BMG Labtech, Ortenberg, Germany). BCECF [2,7-bis-(2-carboxyethyl)-5-(and-6)-carboxyfluorescein],7H4M 7-Hydroxy-4-Methylcoumarin.

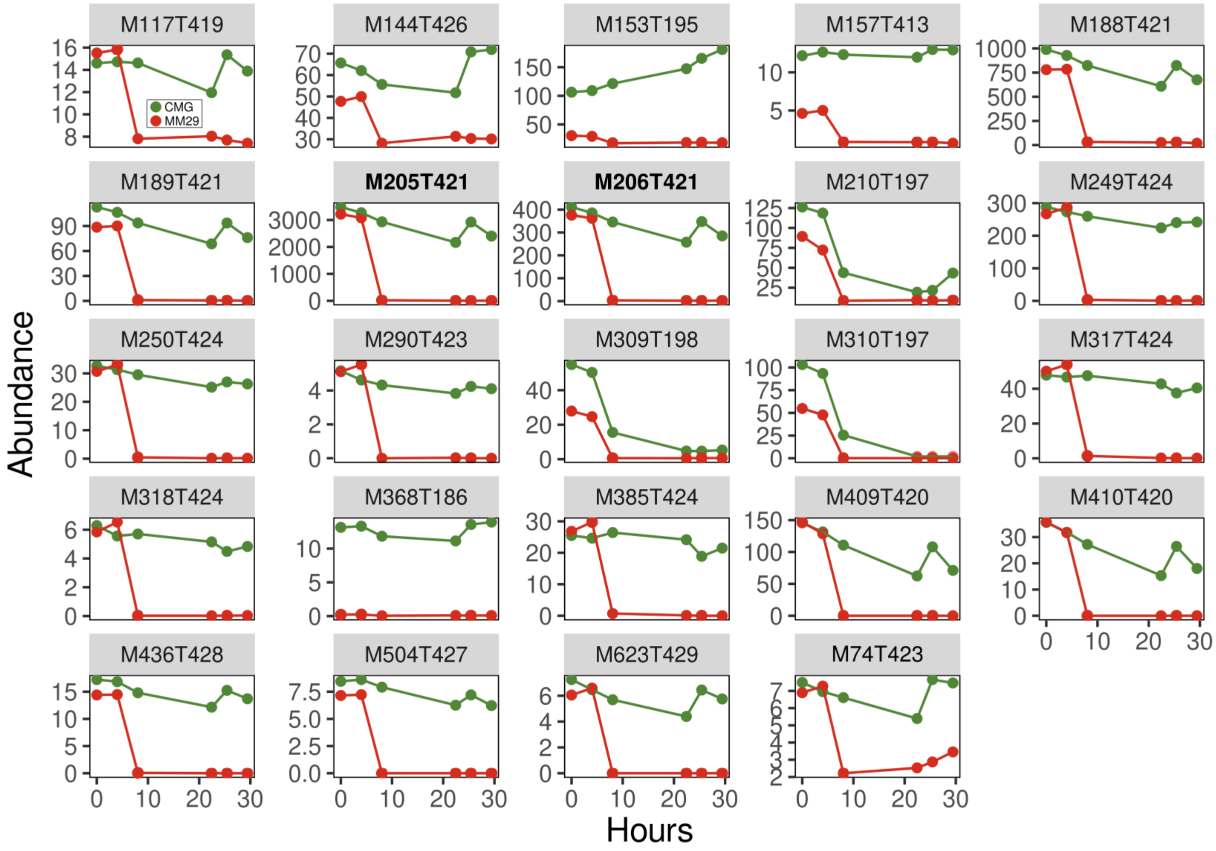

Fig S7 Abundance profiles of the 24 metabolite features in the target cluster. Metabolite abundances were measured by LC-MS for *E. coli* ATCC 25922 cultures growing in either MM29 (red) or CMG (green) medium at six time points (0, 4, 8, 22, 25, 29h) after inoculation. Abundances are based on extracted ion chromatograms. Names of metabolite features are shown above each plot. The two metabolite features identified as tryptophan using the mzCloud database (M205T421 and M206T421) are shown in bold.

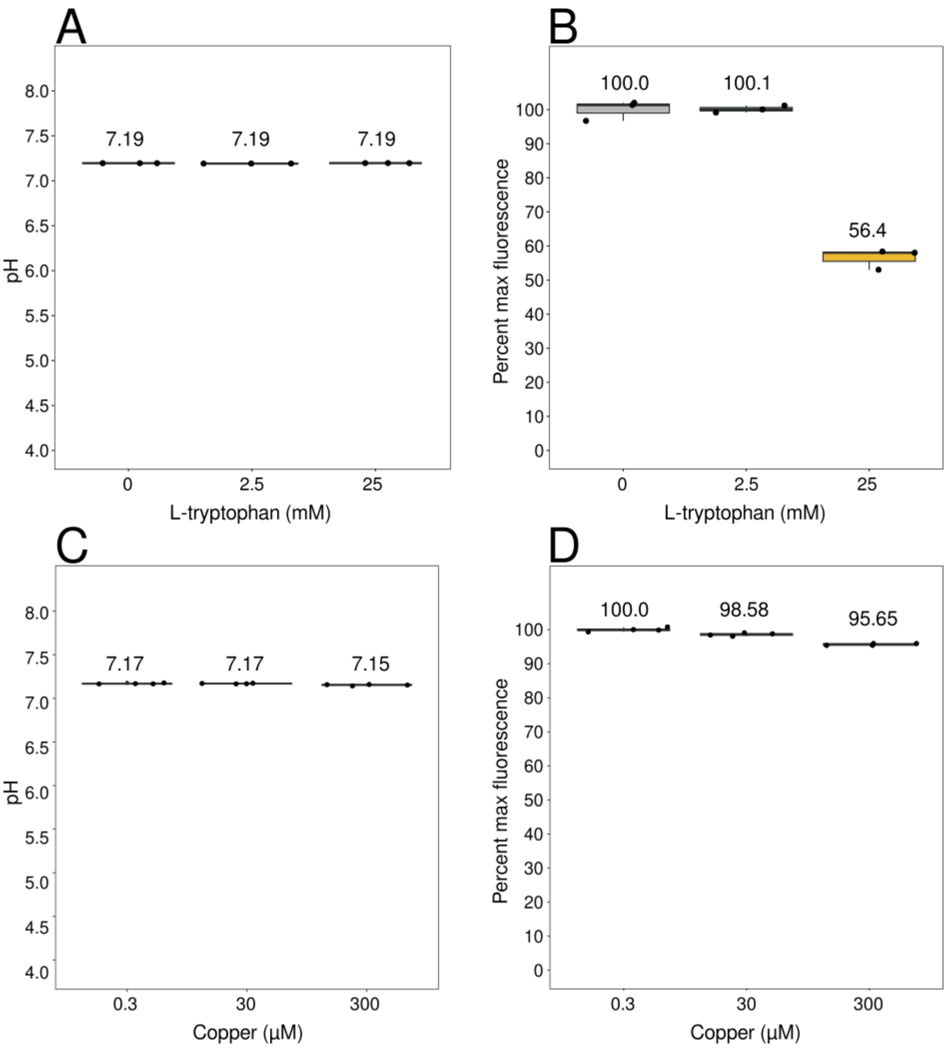

Fig S8 Effects of L-tryptophan and copper chloride on pH and 7H4M fluorescence in MS medium. MS medium was supplemented with 100 µM 7H4M, 2 µM BCECF. The pH was measured as the ratio of the fluorescence of BCECF at the pH-sensitive point (485±15 nm excitation; 540±20 nm emission) relative to the pH-insensitive isosbestic point (450±15 nm excitation; 540±20 nm emission). 7H4M fluorescence was measured as 360±40 nm excitation, 440±20 nm emission and is shown as the percent of fluorescence in standard MS medium (0 mM L-tryptophan, 0.3 µM copper chloride). Box plots show median and interquartile ranges and the mean pH or 7H4M fluorescence shown above data points in each plot. BCECF [2,7-bis-(2-carboxyethyl)-5-(and-6)-carboxyfluorescein], 7H4M 7-Hydroxy-4-Methyl-coumarin.

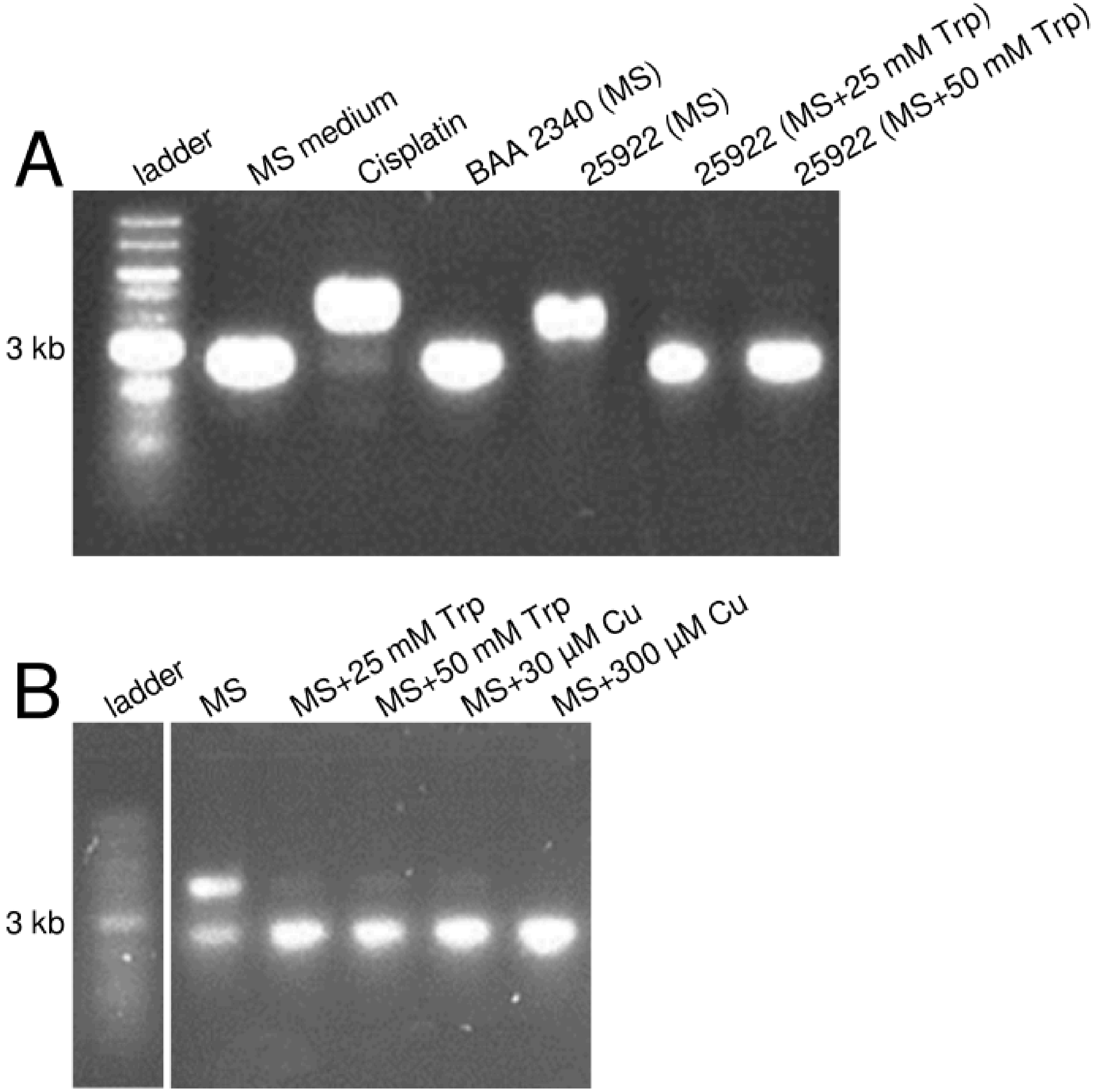

Fig S9 Gels from replicate experiments showing effects of (A,B) L-tryptophan or (B) copper chloride supplementation on DNA crosslinking activity of *pks*+ *E. coli* ATCC 25922 cultures. DNA crosslinking assay performed on *Bam*HI-linearized pUC19 DNA (200 ng per lane) that was incubated for 6h at 37°C with either MS medium, *E. coli* ATCC BAA 2340 (*pks*-) in MS medium, *E. coli* ATCC 25922 (*pks*+) in MS medium with different L-tryptophan or copper chloride concentrations, or 80 μM cisplatin. pUC19 ssDNA is at 2686 bp; pUC19 ICL DNA is at 5372 bp.

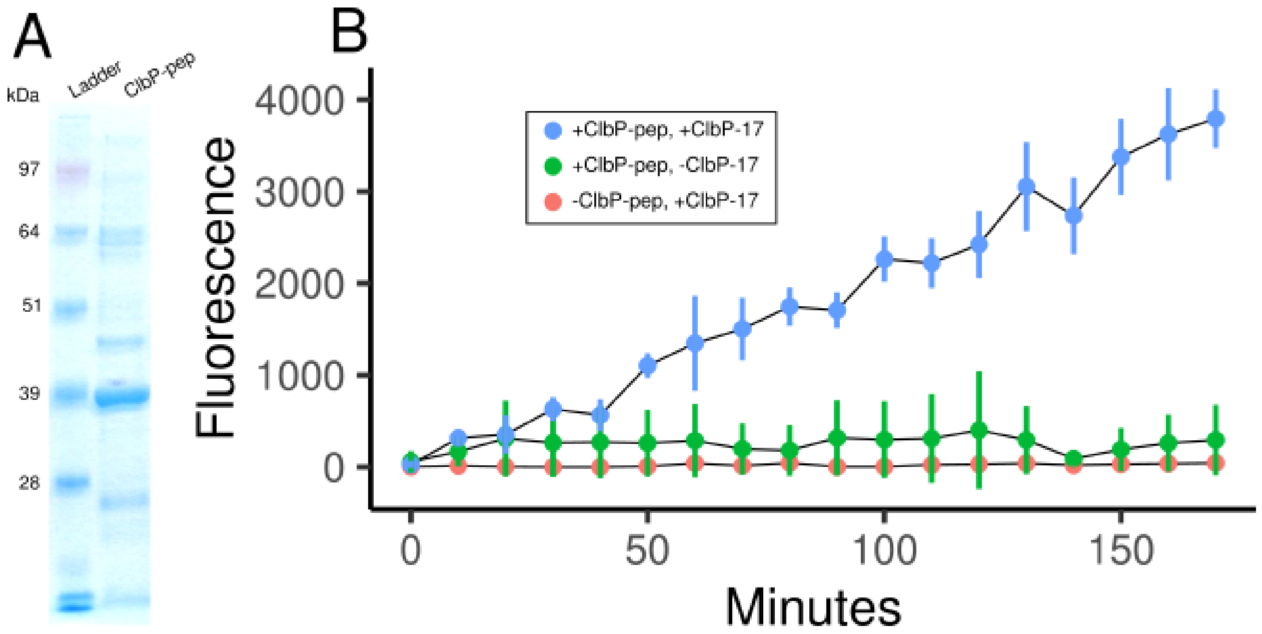

Fig S10 (A) Purification and (B) cleavage of ClbP-17 by the ClbP peptidase domain (ClbP-pep). (A) Partial purification of ClbP-pep was visualized on 12% SDS-PAGE gel (Nupage bis-Tris novex gel IM-8042). Predicted molecular mass of ClbP-pep is 38.3 kilodaltons (kDa). Masses in kDa for the molecular weight ladder are shown to the left of the gel. (B) ClbP-pep was diluted to 0.01 µg μl^{-1} in buffer (50 mM Tris, 200 mM sodium chloride, pH 8) containing 100 µM ClbP-17, as appropriate. Fluorescence (360±40 nm excitation, 440±20 nm emission) was measured using a CLARIOstar microplate reader (BMG Labtech, Ortenberg, Germany).
